# Medial patellofemoral ligament injury patterns and associated pathology in lateral patella dislocation: an MRI study

**DOI:** 10.1186/1758-2555-1-17

**Published:** 2009-07-30

**Authors:** Patrick Guerrero, Xinning Li, Ketan Patel, Michael Brown, Brian Busconi

**Affiliations:** 1 University of Massachusetts Medical Center, Division of Sports Medicine, 55 Lake Avenue North, Worcester, MA, USA

## Abstract

**Background:**

Lateral Patella dislocations are common injuries seen in the active and young adult populations. Our study focus was to evaluate medial patellofemoral ligament (MPFL) injury patterns and associated knee pathology using Magnetic Resonance Imaging studies.

**Methods:**

MRI studies taken at one imaging site between January, 2007 to January, 2008 with the final diagnosis of patella dislocation were screened for this study. Of the 324 cases that were found, 195 patients with lateral patellar dislocation traumatic enough to cause bone bruises on the lateral femoral trochlea and the medial facet of the patella were selected for this study. The MRI images were reviewed by three independent observers for location and type of MPFL injury, osteochondral defects, loose bodies, MCL and meniscus tears. The data was analyzed as a single cohort and by gender.

**Results:**

This study consisted of 127 males and 68 females; mean age of 23 yrs. Tear of the MPFL at the patellar attachment occurred in 93/195 knees (47%), at the femoral attachment in 50/195 knees (26%), and at both the femoral and patella attachment sites in 26/195 knees (13%). Attenuation of the MPFL without rupture occurred in 26/195 knees (13%). Associated findings included loose bodies in 23/195 (13%), meniscus tears 41/195 (21%), patella avulsion/fracture in 14/195 (7%), medial collateral ligament sprains/tears in 37/195 (19%) and osteochondral lesions in 96/195 knees (49%). Statistical analysis showed females had significantly more associated meniscus tears than the males (27% vs. 17%, p = 0.04). Although not statistically significant, osteochondral lesions were seen more in male patients with acute patella dislocation (52% vs. 42%, p = 0.08).

**Conclusion:**

Patients who present with lateral patella dislocation with the classic bone bruise pattern seen on MRI will likely rupture the MPFL at the patellar side. Females are more likely to have an associated meniscal tear than males; however, more males have underlying osteochondral lesions. Given the high percentage of associated pathology, we recommend a MRI of the knee in all patients who present with acute patella dislocation.

## Introduction

Acute lateral patella dislocations are common injury pattern seen in the younger and athletic population that presents to orthopaedic surgeons. A number of them occur as a result of injuries during sports from a traumatic event and some are seen with minor injuries in patients with a history of lateral patella instability. The incidence of acute lateral patella dislocations may account for 2–3% of all knee injuries [1].

The patella is a sesamoid bone within the extensor mechanism that articulates with the distal femur and acts as a fulcrum to transmit large forces from the quadriceps to the tibial tubercle at varying angles of flexion. Because there is a natural 6 degrees valgus angle between the femur and tibia in the coronal plane and a similar angle between the trochlear groove and the tibial tubercle, there is a lateral vector force with the quadriceps contraction acting laterally on the patella. To counteract these forces, the patella has a convex shape with a vertical central ridge and the femoral trochlea has a concave vertical groove with a high lateral wall, particularly through the range where the forces are the greatest [2].

To enhance the static stability, the patella is surrounded by a multilayered system of ligaments that control the movement and position of the patella during force transmission [3]. The three layered system described by Warren and Marshall has been accepted as the correct way to refer to the layers on the medial side of the knee [3]. The superficial layer (layer 1) also called the crural or investing fascia, contributes very little to patellar stability and is conjoined to layer 2 antero-superiorly and antero-inferiorly near the patella [4]. Layer 2 is composed of the superficial medial collateral ligament (MCL), the medial patellofemoral ligament and its oblique decussation from the upper MCL and the patellotibial ligament which is conjoined with layer 1. Layer 3 is composed of the deep MCL, the joint capsule and the medial patellomeniscal ligament [5]. Together, all three layers from the medial retinaculum. On the lateral side, there are interconnecting fascia along with the illiotibial (IT) band, hamstrings, quadriceps and lateral patellofemoral ligament [3]. As the MPFL approaches the patella, it becomes conjoined with the deep portion of the tendon of the Vastus Medialis Oblique (VMO) muscle and together they attach to the upper medial 2/3 of the patella. The VMO is a part of the Vastus Medialis muscle, however, the fibers have an oblique orientation thus giving them the name VMO. Desio et al. compared the soft tissue restrains to lateral patellar dislocation and indicated that the MPFL is the primary stabilizer [5]. Similarly, other studies have found that the MPFL provides anywhere between 50–80% of the stability to prevent lateral patella displacement or dislocation [4-7].

Several studies have been performed evaluating the tear patterns as well as tear location of the medial patellofemoral ligament (MPFL) after patella dislocation. Based on our review of the literature, during lateral patella dislocations, tears have been more commonly reported to occur at the femoral attachment site [8-14]. Due to the fact that in our clinical practice, we have seen what appears to be a greater incidence of MPFL tears occurring at the patellar attachment site with acute patella dislocations, we undertook this retrospective MRI study to assess the injury pattern of MPFL as well as the incidence of associated knee pathology in patients with lateral patella dislocations.

Our hypothesis is that in an event of acute patella dislocation as indicated via bone marrow edema on MRI, the MPFL will likely tear at the patella attachment site with associated injuries patterns in the knee which may include but not limited to meniscal tears, osteochondral lesions, loose bodies, and medial collateral ligament sprain/tears. We further hypothesize that there will be a difference in the incidence of associated pathology between the male and female patient population.

## Materials and methods

This study was performed in accordance to the guideline of the Institutional Review Board of our hospital. A retrospective review of MRI studies of patients with the diagnosis of patellar dislocation between January of 2007 and January 2008 at a single imaging site (1.5 Tesla High Field Open MRI Systems, Framingham, MA) was carried out. To be included in this study of acute patellar dislocation, the MRI had to demonstrate bone marrow edema on the anterolateral femoral condyle and the medial patellar facet. Of the 324 studies screened, 195 MRIs met these criteria. There were 127 males and 68 females with a mean age of 23 years. The remaining 129 MRI studies that did not meet our criteria of acute lateral patella dislocation as evidences by a lack of bone marrow edema were excluded.

Each MRI study was reviewed by three physicians: a musculoskeletal radiologist, a sports medicine fellowship trained orthopaedic surgeon and an orthopaedic sports medicine fellow. The reviews were performed independently and any discrepancies were settled by a consensus within the group. The reviewers looked at the Medial Patella Femoral Ligament (MPFL) in the axial, coronal and sagital views in both T1 and T2 weighted images. Tears, attenuation, avulsion fractures of the MPFL were recorded. Specific location of the tears from its femoral attachment, patellar attachment or both was further documented. Additional findings such as the presence of loose bodies, meniscal tears and osteochondral defects were also evaluated. Statistical analysis was performed with the student t-test method in order to provide statistical significance, which is set at p < 0.05.

## Results

**Figure 1:**
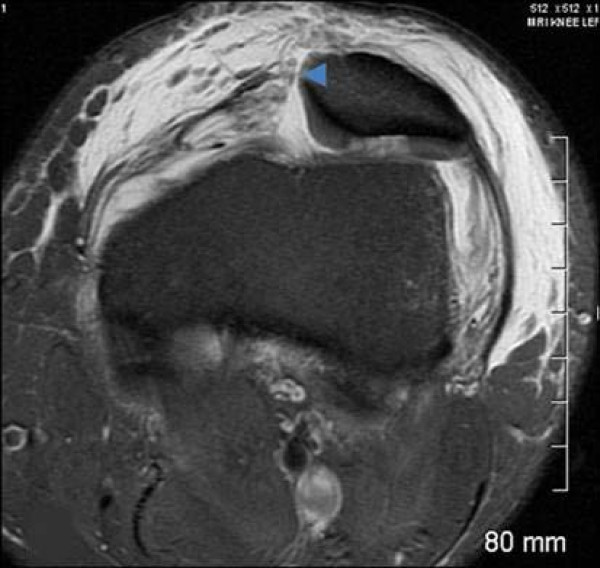
**Axial T2 MRI image displays a tear at the patellar attachment (arrowhead)**.

**Figure 2:**
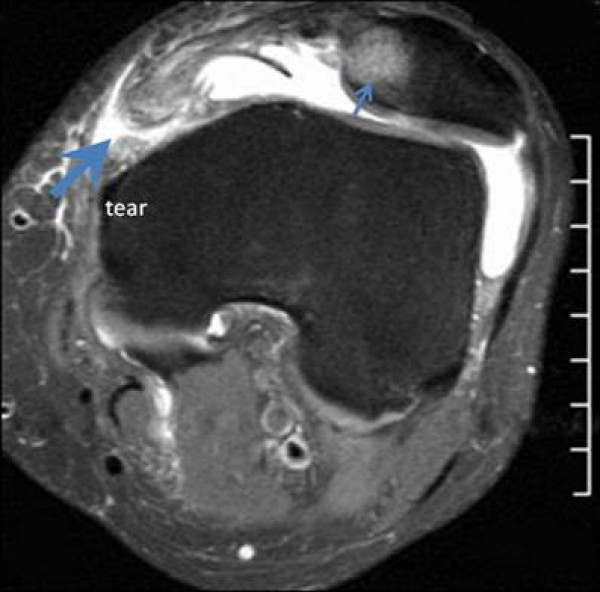
**Axial T2 MRI image indicates tearing at the femoral attachment (large arrow) and bone contrusion at the medial patellar facet (small arrow)**.

**Figure 3:**
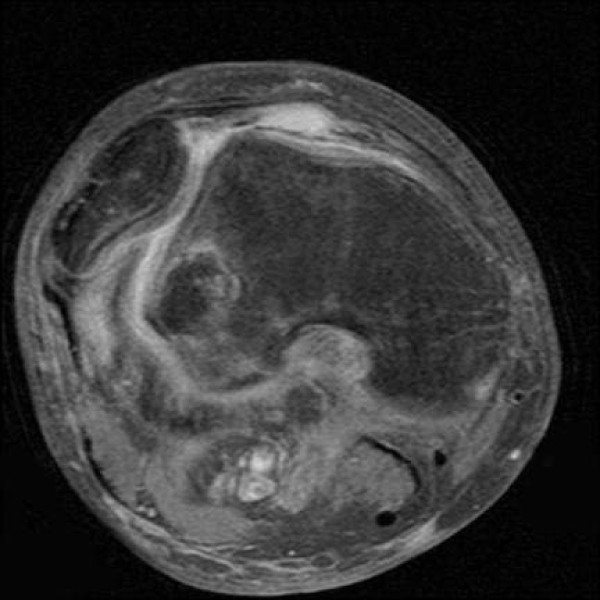
**The MPFL is attenuated without a discrete tear noted with a laterally dislocated patella**.

**Figure 4:**
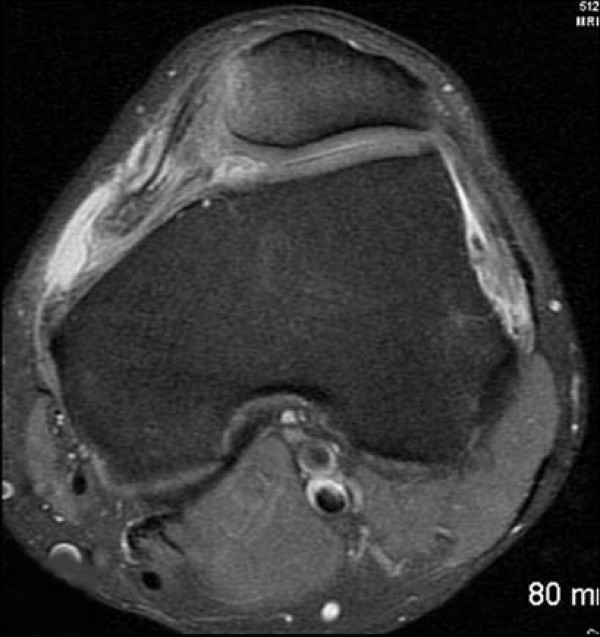
**There is complete avulsion of the MPFL at the femoral attachment and a tear at the patellar attachment**.

**Figure 5:**
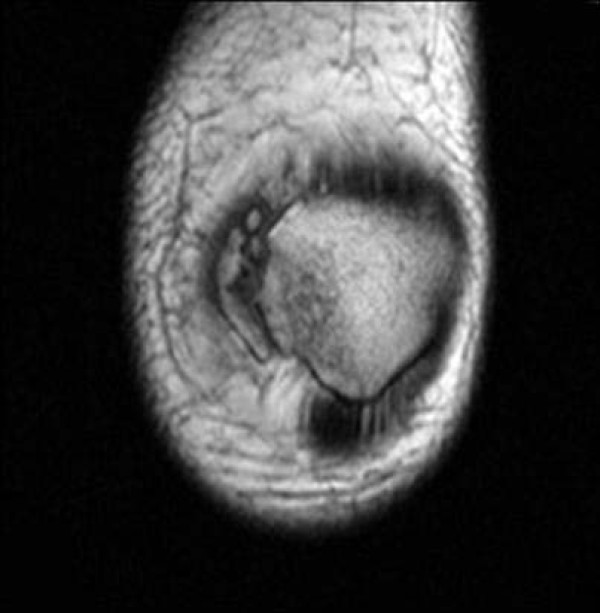
**Avulsion fracture at the medial attachment of the MPFL**.

**Figure 6:**
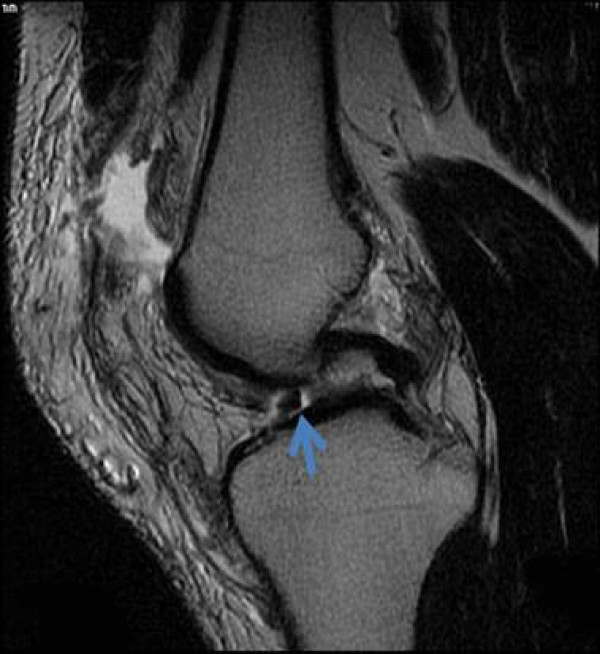
**A loose body at the inter-condylar notch from an osteochondral defect injury after a lateral patellar dislocation**.

This study consisted of 127 males and 68 females; mean age of 23 years, range of 10–56 years. Tears of the MPFL at the patellar attachment (Figure 1) occurred in 93/195 knees (47%), at the femoral attachment (Figure 2) in 50/195 knees (26%), attenuation of the MPFL without rupture (Figure 3) occurred in 26/195 knees (13%), and at both the femoral and patella attachment sites (Figure 4) in 26/195 knees (13%). Associated findings included patella avulsion/fracture (Figure 5) in 14/195 (7%), loose bodies (Figure 6) in 23/195 (13%), meniscus tears 41/195 (21%), medial collateral ligament sprains/tears in 37/195 (19%) and osteochondral lesions (Figure 2) in 96/195 knees (49%).

When the data is separated into female and male patients, there was no statistical significant difference seen between the two populations when comparing the MPFL injury patterns. The MPFL tear was seen at the femoral site in 29% vs. 23% (p = 0.19), the patella site in 47% vs. 47% (p = 0.49), both the femoral and patella site in 14% vs. 12% (p = 0.33), and attenuation of the ligament in 14% vs. 12% (p = 0.34), when comparing the female vs. the male population, respectively. In terms of loose bodies, 8% vs. 13% (p = 0.17) and MCL tears, 19% vs. 18% (p = 0.48) were seen in the female vs. male population. Statistical analysis showed females had significantly more associated meniscus tears than the males (27% vs. 17%, p = 0.04). Although not statistically significant, osteochondral lesions were seen more in male patients with acute patella dislocation (52% vs. 42%, p = 0.08).

## Discussion

The primary passive restraint in patella instability is the Medial Patellofemoral Ligament (MPFL) which accounts for 60% +/- 13% of lateral restraint with the knee at 20 degrees of flexion [5]. Isolated release of the MPFL will result in 50% increase in lateral subluxation of the patella [6]. The MPFL is located anterior to and in a distinct extra-articular layer from the medial joint capsule. The distal border of the VMO muscle attaches along the majority of the proximal medial edge of the MPFL. The average length of the MPFL is 65.2 mm with a range of 56.8 to 77.8 mm [15].

Sallay el al. evaluated a series of 23 patients with acute patella dislocation using MRI and reported tears of the MPFL in the femoral attachment site at the level of the adductor tubercle in 87% (n = 20), 4% (n = 1) had a tear at the patella attachment site and with 9% (n = 2) had attenuation without tear. Other associated pathology included 22% had loose bodies, MCL sprain in 9%, and meniscal tears in 9% [14]. Several authors also support the finding that tears of the MPFL are likely to occur from or near the femoral epicondyle site after acute patella dislocation [8-10,13,16]. Furthermore, Sillanpaa et al. reported an incidence of 57% rupture of the MPFL at the femoral site, 23% in the mid-substance, and 20% avulsion from the patella after a patella dislocation in a series of 46 military recruits [16]. In contrast to these findings, our study show that 26% of patients had a tear at the femoral site, 13% had tear at both the patella and femoral sites, 13% had attenuation of the ligament without tear, and 47% of the patients had tear of the MPFL at the patella attachment site. The finding that the MPFL will likely rupture at the patella attachment site after an acute patella dislocation is also supported by several investigations in the radiology literature [17-20]. When the data was subdivided into male and female population, there was not a significant difference in the distribution of the MPFL injury pattern (Table 1).

**Table 1:** The location of the MPFL tear as a function of the gender (male vs. female). Attenuation of the MPFL was also recorded.

	Femur Tear	Patella Tear	Femur and Patella Tear	Attenuation Of MPFL
Male (N = 127)	30 (23%)	61 (47%)	16 (12%)	16 (12%)

Female (N = 68)	20 (29%)	32 (47%)	10 (14%)	10 (14%)

Total	50/195	93/195	26/195	26/195

Percent	26%	47%	13%	13%

p-value	0.19	0.49	0.33	0.34

Ellas et al. also supported our data in a magnetic resonance imaging (MRI) study of 82 patients with the diagnosis of lateral patella dislocation and found that 76% of medial retinacular/MPFL disruption occurred at its patellar insertion site, 49% occurred at the femoral attachment site, 30% showed injury of the MPFL at mid-substance, and 48% had more than one site of injury [21]. Furthermore, associated injury patterns that included osteochrondral lesions were seen in 70% of patients, 15% had loose bodies in the joint, and meniscal tears were identified in 11% [21]. Sanders et al. also reported a 40% incidence of osteochondral defect to the lateral femoral condyle after transient dislocation of the patella [22]. In this study, we observed 49% overall incidence of osteochondral lesions with 52% seen in the male population and 42% in the female population (p = 0.08). Nomura et al. reported a 96% incidence of articular cartilage lesion of the patella (fissuring, fibrillation, and erosion) in patients with recurrent patella dislocation. The most common site was the central dome [23].

In the current study, there was a 21% incidence of meniscus tears with 17% seen in the male population and 27% in the female population (p = 0.04). It is hard to explain the significant difference in the increased rate of meniscus tears seen with the female patient population, which we believe maybe due to the lower extremity alignment difference or the mechanism of injury, which in the acute setting of a lateral patella dislocation may result in higher risk of meniscal tears in the female population. However, this above statement should be further investigated given that minor meniscal tears seen on MRI may not correlate to actual tears via arthroscopy. Also given the large percentage of patients who have osteochondral lesions in our study with associated meniscal tears, it is hard to discern whether the patients that have the OC lesions are more likely to get a meniscal tear in the setting of a patella dislocation or vice versa. MCL sprain/tears was seen in 19% of our patient population and 17% had patella avulsion fractures. Virolainen et al. also reported concomitant injury to the medial collateral ligament in 25% of their patients with patella dislocation [20]. However, no significant difference was seen in our data when comparing MCL injury between the female and male population (Table 2).

**Table 2:** Other associated knee pathology seen with MPFL injury as a function of gender (male vs. female)

	OCD Lesion	Loose Bodies	Menisci Tears	MCL Tears	Patella avulsion
Male (N = 127)	67 (52%)	17 (13%)	22 (17%)	24 (18%)	10 (7%)

Female (N = 68)	29 (42%)	6 (8%)	19 (27%)	13 (19%)	4 (5%)

Total	96/195	23/195	41/195	37/195	14/195

Percent	49%	13%	21%	19%	7%

p-value	0.08	0.17	0.04	0.48	0.30

MRI findings of MPFL injury pattern have been investigated with surgical correlation in 14 patients and was found to be 85% sensitive and 75% correct in diagnosing injury patterns in the MPFL [22]. Nomura et al. further studied the correlation of MRI findings with open surgical exploration and reported an 81% accuracy rating (21/26 patients correctly diagnosed) [24]. Therefore we believe using MRI is an accurate method to detect injury to the MPFL ligament and associated knee pathology after acute patella dislocation, however, future studies with larger number of patients should be performed to further verify the sensitivity and specificity of MRIs in diagnosing MPFL injury.

The major strength of our study is that this is the first study to evaluate the incidence of MPFL injury location/pattern and also associated knee pathology in a very large number of patients (n = 195) with a diagnosis of acute patella dislocation as evidenced by the bone marrow edema in a given year (Jan, 2007 to Jan, 2008) from a single MRI site database (Framingham, MA). The results were reviewed by three independent reviewers to confirm and agree on the MPFL injury pattern and associated knee pathology. Also, the data was sub-grouped and statistically analyzed with both the male and female population to compare gender differences.

There are several limitations to this study. First, the major limitation is that these patients were not evaluated clinically and therefore we were unable to determine if these are first time acute dislocations or if they were acute dislocations on chronic instability. Therefore, these findings can not be extrapolated to all patella dislocations but only to the subset of patients who present with bone marrow edema on MRI after a diagnosis of patella dislocation. Furthermore, we can not correlate the mechanism of injury to the MPFL injury pattern and associated knee pathology as these patients' medical records were not available for review. Not being able to physically exam the patients also hinders the ability to determine stability of the knee and if their pain location correlates with the MR findings. Secondly, these MRI's were retrieved and reviewed from a computer database, therefore we do not know if any of the patients underwent operation and what the intra-operative findings were to correlate with the accuracy of these MR image findings.

## Conclusion

Patients who present with lateral patella dislocation with the classic bone marrow bruise pattern seen on MRI will likely rupture the MPFL at the patellar side. Other injury patterns include tear at the femoral site, tear from both sites, or attenuation may also be observed. Females are more likely to have an associated meniscal tear than males; however, more males have underlying osteochondral lesions. Knowing the injury pattern or tear location of the MPFL and associated knee pathology will be essential in the management of patients with acute lateral patella dislocation, especially if surgical intervention is planned. Given the high percentage of associated injury pathology seen in these patients who have a lateral patella dislocation, we recommend a MRI scan of the involved knee in all patients who have a patella dislocation.

## Competing interests

The authors declare that they have no competing interests.

## Authors' contributions

XL, PG, MB and BB have contributed to the conception/design, data collection/interpretation, and drafting/revising of the manuscript. XL and PG contributed to the collection and analyzing of the data. PG, MB, and KP contributed to the review of the MRIs. All authors approved the final manuscript.
